# Intestinal Paresis with Special Reference to Pituitrin in Its Treatment*Read before the Aesrulapian Club, Nov. 18, 1915.

**Published:** 1916-07

**Authors:** A. J. Colton

**Affiliations:** Physician to Buffalo Hospital, Sisters of Charity and St. Marys Hospital


### BUFFALO MEDICAL JOURNAL

Yearly Volume 71 JULY, 1916. Number 11

### ORIGINAL ARTICLES

The right is reserved to decline papers not dealing with practical medical and surgical subjects, and such as might offend or fail to interest readers. Contributors are solely responsible for opinions, methods of expression and revision of proof.

### Intestinal Paresis With Special Reference to Pituitrin in Its Treatment.*

By A. J. COLTON, M. D.,

Physician to Buffalo Hospital, Sisters of Charity and St. Marys Hospital.

Intestinal paresis is known by various names, such as intestinal atony, intestinal motor neurosis, intestinal insufficiency, functional debility of the intestinal muscular system, stasis, etc. All of these terms are used to express a condition of the intestinal tract in which there is either a diminution or loss of intestinal peristalsis; or, in other words, it is the partial or complete loss of that force which produces the onward movement of the intestinal contents.

This condition may be partial or complete, chronic or acute, organic or functional.

Without the peristallic function we would be most miserable, short lived and all eventually die as surely as if we suffered from an organic obstruction of the bowels.

Intestinal paralysis I shall consider the absolute loss of muscular power of the intestines and usually due to some disturbance of the cerebrospinal or sympathetic nervous system; organic in nature and in contradistinction to the former condition, viz:—paresis, atony, insufficiency, stasis, etc., which I shall consider functional whatever that may mean. The above enumerated conditions, intestinal paralysis, and any condition producing a mechanical obstruction to the

*Read before the Aesculapian Club, Nov. 18, 1915.

alimentary canal, such as hernia, volvulus, intussusception, tumors, adhesions, etc., are excluded from the discussions of this paper.

According to Boas both the paretic and paralytic conditions are confined to the large intestines only and entirely unknown to the small ones.

Why this broad statement is made by Boas I cannot explain as both the large and small intestines receive their nerve supply from the cerebrospinal and sympathetic nervous systems; although the colon, as far as I am able to determine, receives no innervation from the spinal nerves but is supplied by the sympathetic and ganglionic nervous system, while the balance of the large intestines, viz: the sigmoid and rectum receive branches from the sacral plexus as well as the sympathetic and ganglionic nervous systems.

The small intestines are innervated by the sympathetic and ganglionic nervous system with a communicating branch from the pneumogastric. These unite to form the Auerbach plexus between the circular and longitudinal muscular fibres and from these emerge fibres to the submucous and mucous tissue forming the Meissner plexus.

Both the small and large intestines have the same number of layers, viz: four,—serous, muscular, submucous and mucous. Why, therefore, the condition of intestinal paralysis, paresis, atony, etc., is confined exclusively to the large intestine, I am at a loss to explain. That the condition of paresis, atony, stasis, etc., does exist at times in some portion of the intestinal tract, no physician or surgeon can deny. Neither can it be denied that it is one of grave danger at times as surgeons doing extensive operations on the alimentary canal have experienced.

The chronic condition is one of obstipation and although the immediate effects are not as disastrous as in the acute, the ultimate results are the same.

Among the causes of the chronic type may be mentioned congenital malformations, tight sphincter, constant use of strong cathartics, excessive and frequent use of enemas, (this producing an over-distension of the colon and muscle exhaustion) frequent child bearing causing relaxed abdominal walls, thus losing an important auxiliary force in defecation. Irregular habits, both as to meals and defecation, are causes, as well as improper foods, overfeeding, intestinal indigestion, this producing fermentation and gas formation and thus leading to colon distension and acute dilatation. Neurotic and emotional disturbances are also frequent factors by acting on the inhibitory nerves (the splanchnic).

Arteriosclerosis and Basedow's disease are mentioned as causes, also narcotic and astringent drugs are causative factors.

The symptoms are those of constipation and constipation is a cause as well as an effect of the disease. Other symptoms are sallow skin, anorexia, loss of weight, coated tongue and distended abdomen, gaseous eructations and emotional disturbances.

Its treatment has been as varied as its causation. The first treatment should be the correction of faulty and vicious habits, such as irregular feeding, improper foods and postponing the calls of nature and thereby producing muscular exhaustion and distension. Various forms of electricity to the abdomen and lumbar portion of the spinal cord are recommended and especially the Faradic current.

Massaging of the abdomen and abdominal pressure while lying in bed by the use of a sand bag, also abdominal belts have long been used.

Stretching of a tight sphincter muscle sometimes gives much improvement. Various laxative drugs, such as cascara, podophyllin, aloin, and also drugs acting as cerebrospinal stimulants, such as nux, ignatia, strychnine, etc., are of decided value when combined with some of the former laxatives. A popular and somewhat effectual drug in treating this condition is white liquid paraffin of the Russian oil type.

One of the most effectual means in my hands has been the use of the Bulgarian lactic acid bacillus grown in milk. It is most efficacious in those cases where the paresis is due to an intestinal toxaemia and acts both as a food and drug.

A great deal has been written of late of this condition under the name of intestinal stasis and I believe that Lane was the first to use the term.

Bainbridge compares the intestinal canal when in the condition of stasis to a faulty sewer system in which low points exist, thus producing slowing and stagnation of the stream. To correct this it is now the fad to operate on the colon, lifting up the low points and attaching them to some portion of the abdominal wall or short circuiting the intestine by an intestinal anastomosis, thus overcoming the Lane kinks and other faulty conditions.

Comparing the drainage in the intestinal canal to a sewer system to me has seemed farfetched for if the peristalsis were unimpaired the bowels would move in man or beast in an inverted position, i.e., heels up and head down, thus making all points in the canal low points.

This to my mind disproves the similarity of the intestinal canal to a sewer system.

I now come to the part in the discussion of this paper in which I am most interested and which prompted me to write it, viz: the acute intestinal paresis and its treatment.

The acute paretic condition is one of recent obstipation, having many of the physical and clinical symptoms of mechanical obstruction of the bowels, minus the pain and localized tenderness and the absence of any tumor.

Its causes are almost as numerous as the chronic type and decidedly more serious as to immediate consequences.

Many cases make spontaneous recoveries while others go from bad to worse under the most approved treatment.

Among the various causes of acute intestinal paresis may be mentioned appendicitis, peritonitis, embolism of the mesenteric artery, prolonged, difficult and instrumental deliveries in obstetrics. Traumatism or blows over the solar plexus, operations on the testicle and tapping an inflamed hydrocele may produce it, (probably reflexly) also operations on the tubes and ovaries and prolonged anesthesias.

Roughly handling and undue exposure of the gut during intestinal operations are common causes. In fact this is as great a bugbear to the surgeon as a peritonitis and has been extremely difficult to overcome.

The meteorism in typhoid fever is undoubtedly also one of intestinal paresis due to ulceration, notwithstanding the statement made by Boas that paresis of the small intestine is unknown.

Pneumonia is also not an unusual cause and, by the way, let me digress for a moment.

Not long ago I was called to see a child with lobar pneumonia which had been diagnosed as appendicitis by a local surgeon and he was about to operate, but I insisted that it was a case of pneumonia. The operation was postponed. The abdominal symptoms were those of constipation, abdominal tenderness and distension, but a big dose of castor oil in this case removed the abdominal distension and tenderness.

Three days later there was a crisis followed by a rapid convalescence and recovery.

Not all cases of pneumonia with intestinal paresis respond as readily to castor oil as the following case will show, viz:

Mrs. W. Aet. 46. Sent to hospital with double pneumonia.

On the fifth day of her illness developed obstipation with considerable abdominal distension. With this came increased frequency and labor in breathing and a rise in pulse rate. Cathartics were ordered but no action; again repeated with no result except increased distension and greater difficulty in breathing. Noble's enema was given but the only result was a slight return of the enema.

The abdomen was now greatly distended and tympanitic.

The diaphragm was crowded upwards, thus interfering very much with an already embarrassing condition of the lungs and heart. The breathing became very noisy, labored and difficult. The finger tips and lips were becoming cyanised.

The patient became delirious and the heart rapidly failing although she was getting hypos of camphorated oil and strychnine alternately in connection with one ounce doses of whisky. All treatment seemed to be of no avail and the patient was actually dying. Her blood pressure had dropped 40 points. Her friends had given up hopes and were prepared for the worst.

We had about exhausted our medical resources but as a last means, owing to the sudden dropping of the blood pressure and recollecting the effect of Pituitrin on raising blood pressure, also the effect that it has when given to parturient women in producing a desire to go to stool, we gave 1/2 c.c. of Pituitrin very slowly into the median cephalic vein. Within 30 seconds there was the most remarkable effect I ever witnessed of any drug on a human being, viz: the expulsion of large quantities of flatus with a large liquid stool that nearly filled the bed pan. The abdomen in less than three minutes was down to its normal condition. The only thing to which I could liken it was the deflating of an auto tire after removing the valve, so rapidly did the abdominal distension subside.

The frequent and labored respiration began to lessen immediately. The heart's action picked up and the cyanosis rapidly disappeared.

In fact her convalescence started from the moment of her intravenous injection and in ten days she left the hospital practically well.

Since using this I have learned that in two Pittsburgh hospitals it is a standard treatment after all cases of abdominal section and that no intestinal paresis has occurred since its use was instituted.

In looking up medical literature I find that Bell and Hix recommend liquid Pituitrin in intestinal paresis. Bidwell also reports the use of Pituitrin in laparotomy cases. Beginning twelve hours after the operation he gives 1/2 c.c. every four hours for three days intramuscularly and from his results he considers it evident that Pituitrin has a very marked effect on the muscular coats of the bowels and that it is able to overcome the temporary paralysis due to their exposure at the time of operations. All patients passed flatus freely within a few hours of the first injection and were free from abdominal pain or distension.

The pulse rate remained much lower than usual and after some of the severest operations the pulse did not go above 80.

I feel certain that if Bidwell had given his Pituitrin intravenously he would not have had to wait an hour for the passing of flatus.

Cushing states that the liquid extract of the posterior lobe of the pituitary body acts directly on the involuntary muscles, such as those of the heart, uterus, stomach, intestines, etc.

He gives various other actions on different organs, but these have no relation to the subject under discussion.

I make no claim as to original discovery of the stimulating action of Pituitrin on the muscle fibres of the intestine, although as far as I know I am the first to use it intravenously for that purpose.

Dr. W. H. Park, director of the Research Laboratories of the New York Health Department, states that an injection of diphtheria antitoxin administered intravenously will act in one-tenth of the time than when given subcutaneously. This he has proven by the Schick test. Why then is it not fair to assume that Pituitrin acts the same way?

Bidwell administered Pituitrin intramuscularly for intestinal paresis and gets results in about one hour. We administered it intravenously and got results in 30 seconds.

In the acute intestinal paresis I believe we have in Pituitrin a most valuable drug and will prove a life saver in a great many of these conditions.

Since reading the above paper I met with a case that did not respond to Pituitrin intravenously, but the cause was very evident as the following will show, viz:—

Mrs. K., operated on for removal of gall bladder with extensive dissections for adhesions. Patient did well up to third day when pulse began to go up with slight rise of temperature. Abdominal distension and no bowel movements. 1 c.c. Pituitrin was administered intravenously but the usual prompt expulsion of flatus and stools were not in evidence. Pulse became more rapid and distension greater, but not nausea or vomiting.

A second operation was made and there was found 42 in. of gangrenous gut due to embolism of mesenteric artery and which was resected.

27 Jewett Avenue.

---

**Imperforate Hymen.** Herbert R. Spencer, Lancet, April 15, advocates routine examination and prompt rupture with scissors, using oiled gauze. He reports a case. His argument is mainly on account of the serious troubles arising at about the age of puberty.

